# Household-specific barriers to citizen-led flood risk adaptation

**DOI:** 10.1038/s44168-024-00198-y

**Published:** 2024-11-25

**Authors:** Ben C. Howard, Cynthia A. Awuni, Samuel Agyei-Mensah, Lee D. Bryant, Alexandra M. Collins, Sandow Mark Yidana, Gerald A. B. Yiran, Wouter Buytaert

**Affiliations:** 1 Department of Civil and Environmental Engineering, Imperial College London, London, UK; 2 Department of Geography and Resource Development, University of Ghana, Ghana, Ghana; 3 Department of Hospitality and Tourism Management, Tamale Technical University, Tamale, Ghana; 4 Department of Architecture and Civil Engineering, Centre for Climate Adaptation and Environment Research (CAER), University of Bath, Bath, UK; 5 Centre for Environmental Policy, Imperial College London, London, UK; 6 Department of Earth Science, University of Ghana, Accra, Ghana

**Keywords:** Climate-change adaptation, Climate-change policy, Hydrology, Psychology and behaviour, Governance

## Abstract

Adaptation is essential to mitigate the effects of climate change, such as increasing flood risk. In response to widespread maladaptation, citizen-led approaches are increasingly championed, whereby people on the frontline of climate change determine their own objectives and strategies of adaptation. Enabling equitable and effective citizen-led adaptation requires an understanding of the barriers for different groups of people but this is currently lacking, especially in low- and middle-income countries. Using responses to a co-produced household survey (*n* = 286) in Tamale, Ghana, we show that barriers to citizen-led adaptation interventions (*n* = 11) differ between households which we relate to important components of adaptive capacity. Overall, awareness, education, and networks are the most important barriers, but resources and time are important for poor households of fewer members. Barriers also differ between interventions and overall structural interventions are preferred over behavioural. This work can inform policies and actions to support effective and equitable citizen-led adaptation.

## Introduction

Adaptation is essential to mitigate the effects of climate change^1^. For instance, it can reduce flood risk even in the face of climate-driven increases in the magnitude and frequency of flood hazards, a phenomenon known as the ‘adaptation effect’^2^. The necessity to adapt to climate change, especially in low- and middle-income countries where climate risk is concentrated, is now globally recognised^3^. As such, financial support to accelerate adaptation is increasing, e.g., pledges to the Fund for responding to Loss and Damage following the 28th Conference of the Parties (COP28) total US$700 million (of a US$100 billion target)^4^.

Despite motivation and funding, effective adaptation remains a challenge. Most adaptation to date has been conducted using a top-down approach, whereby decisions are made by external actors; however, this approach is increasingly criticised for failing to achieve efficacy and equity in outcomes^5,6^. Conversely, citizen-led adaptation—also known as bottom-up, community-based, or autonomous adaptation—is put forward as the optimal approach, allowing people on the frontline of climate change to determine their own objectives and strategies of adaptation^6,7^. Not only can citizen-led approaches put power and agency in the hands of communities, but they may be more effective because they can be based on local and indigenous knowledge, address locally salient drivers of risk, and avoid unacceptable trade-offs and limitations^8^.

Whilst top-down adaptation typically occurs on the national, regional, and/or city scale, citizen-led adaptation more commonly occurs on the community or household levels^9^. Adaptation at this scale typically reduces risk by limiting exposure of the household to flooding and associated risk accumulations (e.g., disease or building collapse) or by reducing the vulnerability of households to the impacts of flooding. Whilst there are limits to the extent of risk reduction that household-level adaptation can provide, it could offer a direct, scalable, and quickly deployable pathway by which to accelerate resilience building, especially in low- and middle-income countries where larger-scale adaptation can be challenged by institutional and governance limitations^10,11^.

Citizen-led adaptation is increasingly championed in policy and practice but relatively little is known about its effects on risk, its distribution, or the barriers that limit its uptake. Unequal access to these interventions is likely due to differences in adaptive capacity, i.e., the ability to change one’s environment or behaviours in order to better cope with new, different, or more variable and a larger range of conditions^12,13^. Adaptive capacity is an important control of access to adaptation and, therefore, influences the equity in its distribution and outcomes^14^. Citizen-led adaptation may be especially vulnerable to inequitable outcomes because, without top-down facilitation, its distribution is likely to closely follow trends in adaptive capacity which can be highly heterogeneous even within communities^6,15^. Inequitable access to flood adaptation widens profound inequalities by creating ‘adaptation gaps’ and violates the central agendas of the Sustainable Development Goals and the Sendai Framework (e.g., ‘leave no one behind’), ultimately representing maladaptation^1,16^.

Top-down facilitation could help ensure the equity and accelerate the rate of citizen-led adaptation, e.g., in the form of policy, finance, and tools and frameworks of practice^6^. However, this assistance must be founded on a solid understanding of the obstacles that hinder the planning and implementation of adaptation practices, referred to here as ‘barriers’^17,18^. A ‘seemingly endless’ list of possible barriers to adaptation have been identified, including financial constraints, risk perceptions and poor governance^17,19,20^. However, most research has focussed on top-down approaches to adaptation that are implemented on national and city scales, and primarily in high-income countries^9^. It is critical to understand the barriers to household-level, citizen-led adaptation in multiple contexts to inform facilitation programmes so that they can better target barriers for diverse groups, including vulnerable and marginalised households which are often neglected^1^.

Adaptation to flooding is important in many parts of the world, but particularly in sub-Saharan Africa where 74.7 million people are both exposed to flooding and living in extreme poverty, and where the rate of adaptation is slow, thus representing a global hotspot of flood risk^3,21^. In this study, research is conducted in Tamale, the capital of the Northern Region of Ghana (Fig. 5) with approximately 375,000 inhabitants^22,23^. Tamale, like many secondary cities in low- and middle-income countries, is characterised by fast urbanization, chronic underinvestment, and limited planning, including on climate change adaptation. It is increasingly subject to pluvial and fluvial flooding which has significant negative impacts on the health and wellbeing of its residents, many of whom are highly vulnerable^24^. Here, Tamale provides a platform to investigate how citizen-led adaptation is presently distributed, identify barriers to adaptation for different groups, and propose pathways to instigate and empower citizen-led adaptation to flooding.

## Results

### Barriers to citizen-led adaptation

**Fig. 1:**
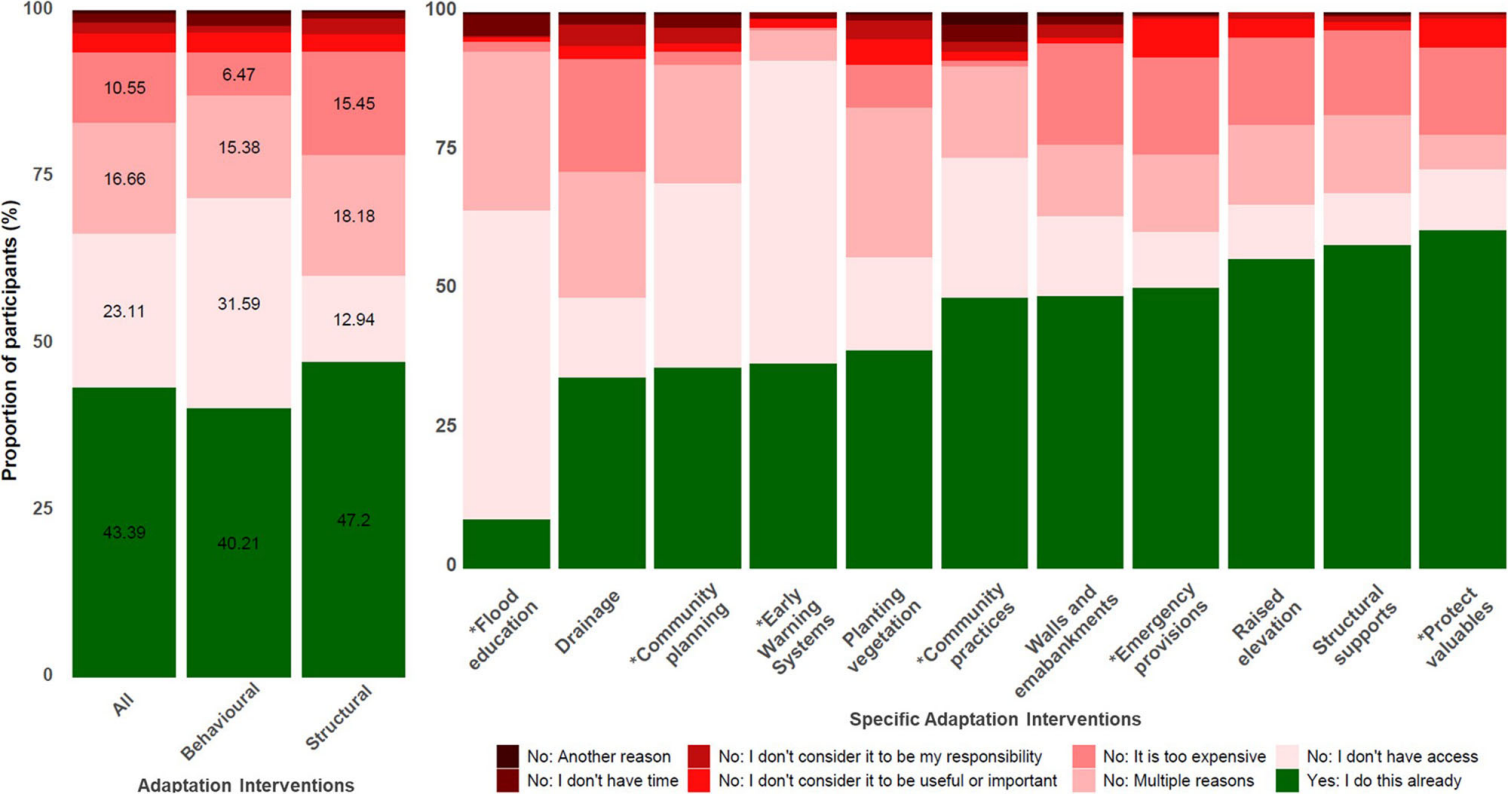
Average rates of practice and barriers to citizen-led adaptation interventions. Left panel: Average household responses to all, behavioural, and structural adaptation interventions. Right panel: Average household responses to individual adaptation interventions. Behavioural interventions are indicated by * symbol; structural interventions (e.g., drainage) are unmarked.

The survey questions about barriers to citizen-led adaptation were dominated by four responses (‘*Yes: I do this already’, ‘No: I don’t have access’, ‘No: Multiple reasons’* and ‘*No: It’s too expensive’*) which together represented 93.7% of all responses. *‘Yes: I do this already,’* indicating existing practice of the intervention (i.e., absence of a barrier), was reported on average by 43.4% of households across all interventions (Fig. 1). *‘Yes: I do this already’* was reported less for behavioural interventions (40.2%) than structural (47.2%), suggesting more structural interventions are currently practiced than behavioural in Tamale.

*‘No: I don’t have access’* was the most reported barrier on average across all interventions (23.1%), followed by *‘No: Multiple reasons’* (16.7%) and *‘No: it’s too expensive’* (10.6%), together representing 88.9% of all responses of barriers (i.e., excluding *‘Yes: I do this already’* responses). This suggests that most households believe that the suite of adaptation interventions we tested are useful and important, that it is their responsibility to practice them, and that they have sufficient time to practice them. *‘No: Another reason’* was only selected on average 0.4% of responses, suggesting that the barriers we offered were salient. Substantial differences were reported in the barriers to behavioural and structural interventions. *‘No: I don’t have access’* was by far the largest barrier to behavioural interventions (31.6%), with ‘No: Multiple reasons’ (15.4%) and *‘No: it’s too expensive’* (6.5%) receiving a lower proportion of responses. In contrast, *‘No: it’s too expensive’* was reported more for structural interventions (15.5%), with a similar proportion of responses received for *‘No: I don’t have access’* (12.9%) and *‘No: Multiple reasons’* (18.2%).

Most adaptation interventions received a similar proportion of the *‘Yes: I do this already’* response, approximately 30-50%. An exception is flood education, to which only 25 households (8.7%) responded *‘Yes: I do this already’*. *‘No: I don’t have access’* was overwhelmingly the primary barrier to flood education whilst *‘No: it’s too expensive’* was hardly a barrier whatsoever. Protecting valuables was the most practiced intervention, with 60.8% of households responding *‘Yes: I do this already’*. Interestingly, the biggest barrier to protecting valuables was *‘No: it’s too expensive’*, which could indicate that those households cannot afford valuables or that protecting valuables is prohibitively expensive relative to their worth.

There are similar patterns in the most common barriers for most interventions. Notable deviations include community planning and community practices, which both received a higher proportion of the *‘No: I don’t have access’* response, 33.2% and 25.2% respectively. Using early warning systems to inform decisions was dominantly hindered by a lack of access (*‘No: I don’t have access’*) (54.5%), and *‘No: It’s too expensive’* was hardly selected at all. Emergency provisions, protecting valuables, and planting vegetation were classified by *‘No: I don’t consider it to be useful or important’* more so than other interventions, 7.0%, 5.2%, and 4.5% respectively, although these still represented relatively small proportions of the total barriers.

### Different households have different levels of adaptation

**Fig. 2:**
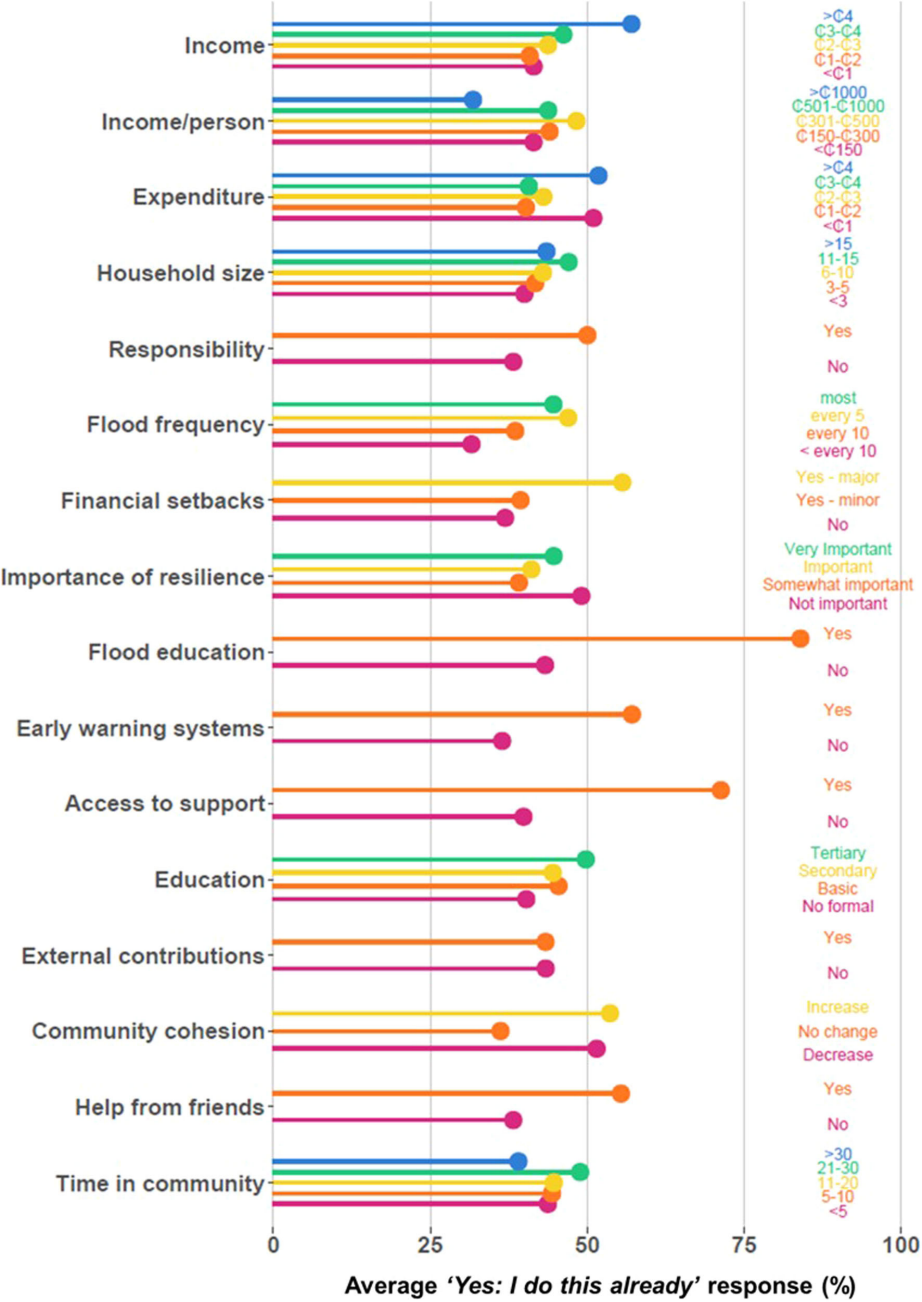
Differences in average practice rates of citizen-led adaptation interventions between classes of household indicators of adaptive capacity. Household characteristic indicators (as classified in Table 3) are plotted against the average ‘Yes: I do this already’ (i.e., no barrier to adaptation intervention) response for each class. Income and expenditure units (₵) represent thousand Ghanaian Cedis. Flood frequency and time in community classes are shown in years.

Households characterised by different demographics (Fig. 6) reported differing levels of adaptation, as shown by variations in the average *‘Yes: I do this already’* response indicating absence of a barrier(s) to specific interventions (Fig. 2). Based on these data, the demographic indicators that had the greatest influence on household practice of flood adaptation interventions include flood education (no = 43.3%, yes = 84.0%), access to support (no = 39.9%, yes = 71.3%) and early warning systems (no = 36.5%, yes = 57.1%). Whilst less pronounced, increased income, feeling of responsibility, experiencing major financial setbacks, access to (general) education, and help from friends also seem to support practice of interventions (Fig. 2).

Some indicators showed clear relations with levels of adaptation. For example, the number of adaptation measures adopted for financial setbacks increased between *‘No, I haven’t experienced setbacks,’ ‘Yes, I have experienced minor setbacks’* and *‘Yes, I have experienced major setbacks.’* Similar positive relationships were observed for income, household size, flood frequency and education where the higher the class (i.e., higher rank in ordinal categorical indicators or *‘yes’* in nominal indicators), the higher the level of adaptation. These relations were not necessarily linear, however, and some exhibited more complex patterns such as threshold effects, i.e., an extremity class performs differently from the others. For example, for income, households in the first four classes (<₵1000, ₵1000-₵2000, ₵2000-₵3000, ₵3000-₵4000) on average responded *‘Yes: I do this already’* between 41% and 47% of the time. Households in the highest class (>₵4000) did so 57.1% of the time, suggesting an important change beyond >₵4000 income. On the contrary, households in the >₵1000 class of income/person showed a much lower *‘Yes: I do this already’* response than the other classes. For duration of time spent in the community, the second highest class (21–30 years) reported the highest average *‘Yes: I do this already’* response whilst the highest class (>30 years) reported the lowest, suggesting that the longest serving community members have the lowest level of adaptation. Other complex patterns include where both the highest and lowest classes show the highest *‘Yes: I do this already’* responses, which was observed for expenditure, importance of resilience, and community cohesion.

### Primary barriers differ between groups

**Fig. 3:**
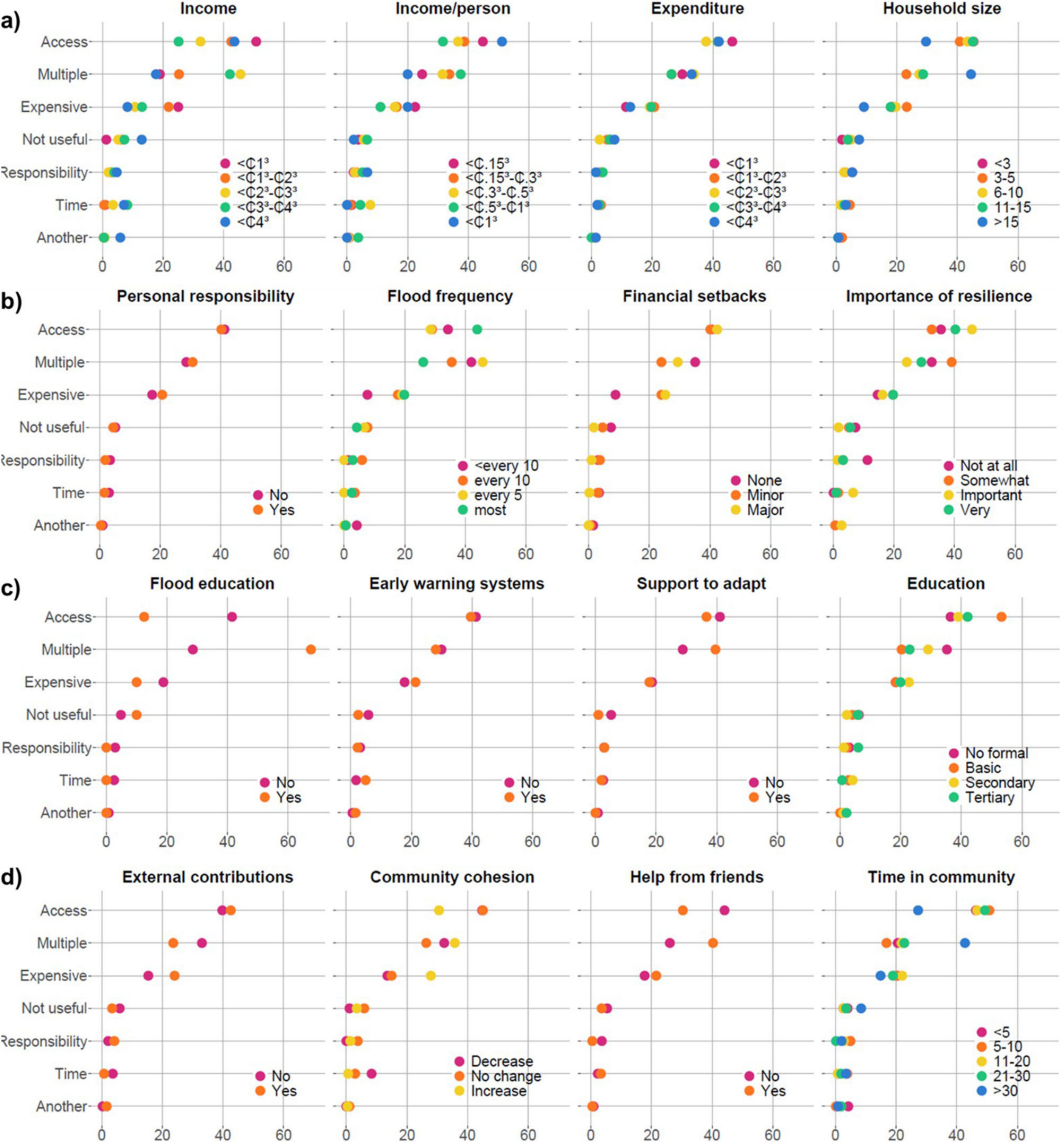
Differences in barriers to citizen-led adaptation interventions between classes of indicators of adaptive capacity. The average percentage response of each self-reported barrier by different classes of indicator across all interventions, grouped by component as outlined in Fig. 6. The four components are presented in panels (**a**) resources and time, (**b**) motivation and responsibility, (**c**) awareness and education, (**d**) social networks.

Household adaptive capacity indicators influence which barriers most hinder the practice of the adaptation interventions addressed, as shown by differences in the proportion of barriers the households in each class reported on average (Fig. 3). Some indicators showed minimal differences between classes, such as feelings of personal responsibility or early warning systems (Figs. 3b and 3c, respectively), which suggests that the barriers are the same regardless of these indicators. However, some indicators showed substantial differences between classes, such as income and household size (Fig. 3a) and flood education (Fig. 3c), which suggest that these indicators control which barriers are important.

Indicators pertaining to resources and time (Fig. 3a) generally showed substantial differences between classes, with the exception of expenditure. For income, *‘it’s too expensive’* was the greatest barrier for the poorest class (<₵1000) and the least prohibitive barrier for the richest class (>₵4000). The richest classes (₵3000–₵4000 and >₵4000) reported that *‘I don’t have time’* was more of a barrier, and the richest class (>₵4000) also cited *‘another reason.’* As in Fig. 2, the lowest and highest class of income/person showed similar patterns. Households of >15 people reported *‘I don’t have access’* as a barrier less than other classes, instead citing *‘multiple reasons’* and *‘I don’t consider it to be useful or important,’* potentially hinting at a diversity of skills, perspectives, and networks within the household.

Motivation and responsibility indicators (Fig. 3b) generally showed less difference between classes than resources and time. Flood frequency did not seem to influence barrier type in any discernible pattern, except that households that were flooded less than every 10 years indicated *‘another reason’* more than the other classes. Households that had not experienced financial setbacks due to flooding generally suggested that *‘It’s too expensive’* was less of a barrier and *‘I don’t consider it to be useful or important’* was more critical.

Awareness and education indicators (Fig. 3c) varied in the differences between classes. Flood education showed the biggest difference between classes, where households who had engaged with flood education reported *‘I don’t have access’* as a barrier only 12.5% on average, whereas those who had not engaged reported it 47.5% on average. In contrast, households who had engaged in flood education reported *‘multiple reasons’* 67.5% on average, compared to 28.6% for those who had not.

All of the social network indicators (Fig. 3d) showed some differences between classes in the most reported barriers. Households that received external contributions cited *‘it’s too expensive’* more than those who did not. Households who observed increases in community cohesion following flooding reported *‘I don’t have access’* as a barrier less frequently but *‘it’s too expensive’* more than those who observed no changes or decreases. *‘I don’t have access’* was cited less frequently by households whose head had lived in the community for >30 years, but *‘multiple reasons’* was cited more frequently.

### Adaptive capacity influences the practice of adaptation interventions

**Fig. 4:**
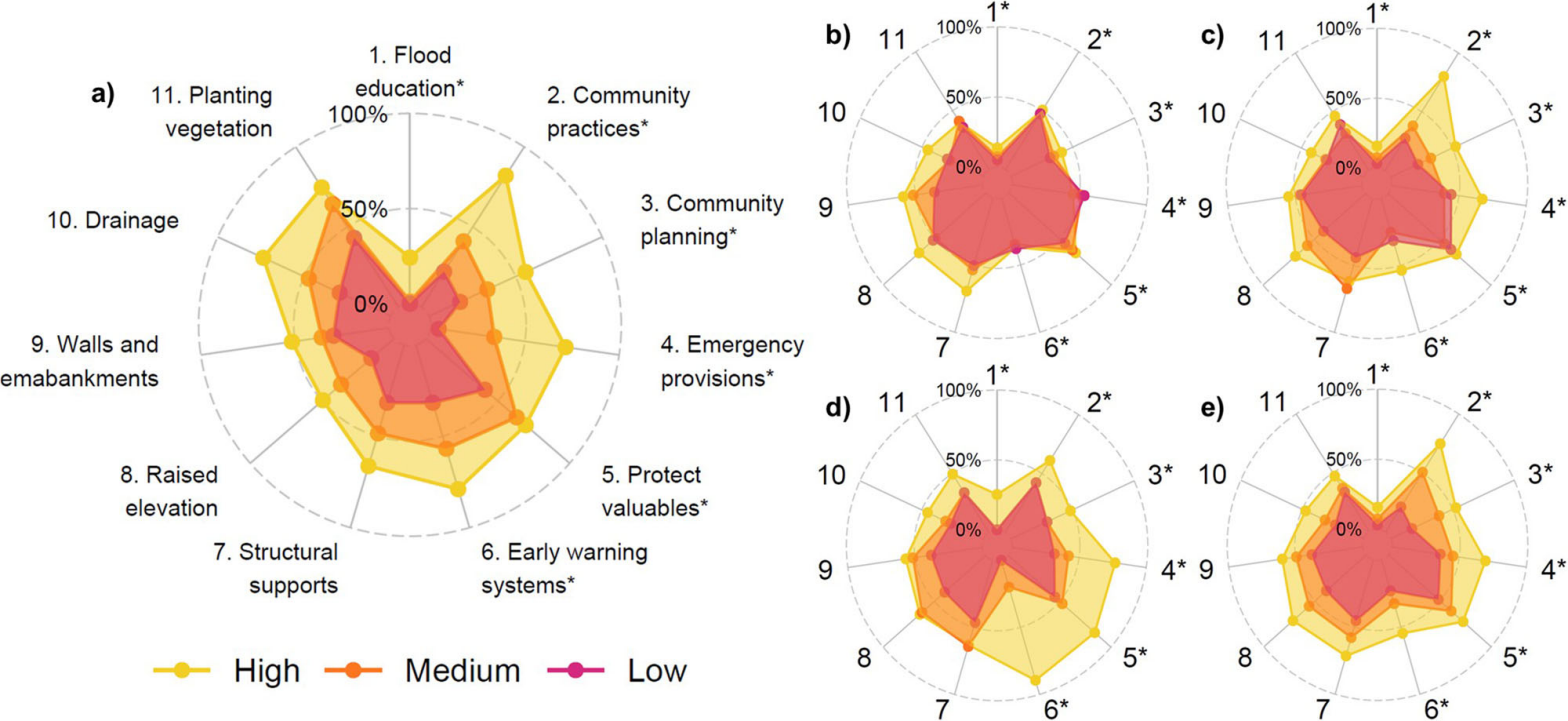
Differences in relations between citizen-led adaptation interventions and adaptive capacity and its components. Low, medium, and high refers to 1st, 2nd, and 3rd tertiles, respectively, of the adaptive capacity index (**a**) and its four components: (**b**) resources and time, (**c**) motivation and responsibility, (**d**) awareness and education, and (**e**) social networks. Behavioural interventions indicated by * symbol.

The proportion of households that practiced each adaptation intervention (Fig. 1) is reflected in corresponding radar plots (Fig. 4). From these results, it is evident that household adaptive capacity affected the practice of some interventions more than others. Households with a high adaptive capacity on average practiced every intervention more than those with a medium adaptive capacity, and households with a medium adaptive capacity practiced every intervention more so than those with a low adaptive capacity (Fig. 4a). The average difference between groups (high, medium, low) was larger between high and medium for behavioural interventions (26.8% compared to 17.4%) and between medium and low for structural (14.9% compared to 18.3%). Furthermore, four of the top five largest average differences between high to medium and medium to low adaptive capacity were exhibited for behavioural interventions (community practices, early warning systems, emergency provisions, and community planning). Combined, these results suggest that a higher adaptive capacity was necessary to enable behavioural interventions as compared to structural interventions.

Awareness and education (Fig. 4d) had the largest effect on the practice of interventions, particularly behavioural. Resources and time (Fig. 4b) had the smallest influence on the practice of interventions overall, with low, medium, and high classes showing similar levels of adaptation. Structural interventions were more affected than behavioural, showing higher levels of adaptation in the high resources and time group as compared to medium and low. In contrast, motivation and responsibility (Fig. 4c) had a larger influence on behavioural interventions, excluding the protection of valuables which showed a similar level of practice for all groups. Similarly, awareness and education (Fig. 4d) had a larger influence on behavioural interventions, where the high group deviated from the medium and low groups. For the structural interventions, however, the medium group was more closely aligned with the high; the low group exhibited lower levels of practice. The social networks component (Fig. 4e) showed the most consistent patterns in its effects on levels of adaptation, exhibiting somewhat linear positive relationships across the groups (low, medium, and high).

## Discussion

This study represents one of the first attempts to understand the barriers that hinder citizen-led adaptation to flooding in low- and middle-income countries. We investigate how (i) the barriers to adaptation are influenced by household characteristics, particularly by important components of adaptive capacity, and (ii) these barriers differ between different adaptation interventions, particularly between structural and behavioural interventions. Understanding this information is central to targeting adaptation strategies for effective and equitable implementation.

In Tamale, Ghana, eleven citizen-led interventions are practiced on average by almost half of the households we surveyed. Individuals and communities on the frontline of climate change are acutely aware of its impacts; as people with agency, a grounded understanding of local system dynamics, and a deep investment in the outcomes, it is no surprise that they respond via adaptation^25^. Recent studies have shown that households can be the most prominent actors implementing adaptation^10^, and practice rates of citizen-led adaptation similar to or even higher than observed in this current study have been demonstrated widely^26–30^. However, stocktakes and assessments of adaptation struggle to include citizen-led adaptation^9,31,32^, not least because it is challenging to measure and monitor and is frequently dynamic, e.g., changing behaviour in response to a recent flood event^33^. Overall, this likely leads to an underestimation of the extent of citizen-led adaptation globally, with potential implications for global climate risk estimates^10^.

Where adaptation is arranged without sufficient participation of the communities who are affected, it is commonly hindered by a lack of interest, motivation, or responsibility, as has been observed for both top-down and community-based approaches^5,34^. In Tamale, very few households thought that interventions were not useful and important, or not their responsibility to practice. Citizen-led adaptation, especially on a household level, may not be hindered by such barriers because the interventions are inherently self-selected and motivated. This may be especially true in cases where citizens’ expectations of authorities to act on their behalf are low^35^. In this study, most households were motivated to practice adaptation but were hindered by other barriers.

The main barriers pertained to knowledge, skills, and networks, as demonstrated in both the self-reported barriers *(‘I don’t have access’*) (Figs. 1, 3) and the adaptive capacity components (awareness, education, and social networks) (Figs. 2, 4). These barriers include not knowing how to practice an intervention or where to access the necessary materials, people, or tools to do so. Similar barriers have been identified in other studies of citizen-led adaptation in different contexts^6,27,34–36^. Overcoming these barriers, e.g., by supporting flood education programmes, fostering feelings of responsibility, or encouraging community and collective action, has been suggested to mobilise behavioural change and scale-up adaptation^27,37,38^. This is supported by our results which show that households who had engaged with these activities were up to more than twice as likely to practice an adaptation intervention (Fig. 2).

**Fig. 5:**
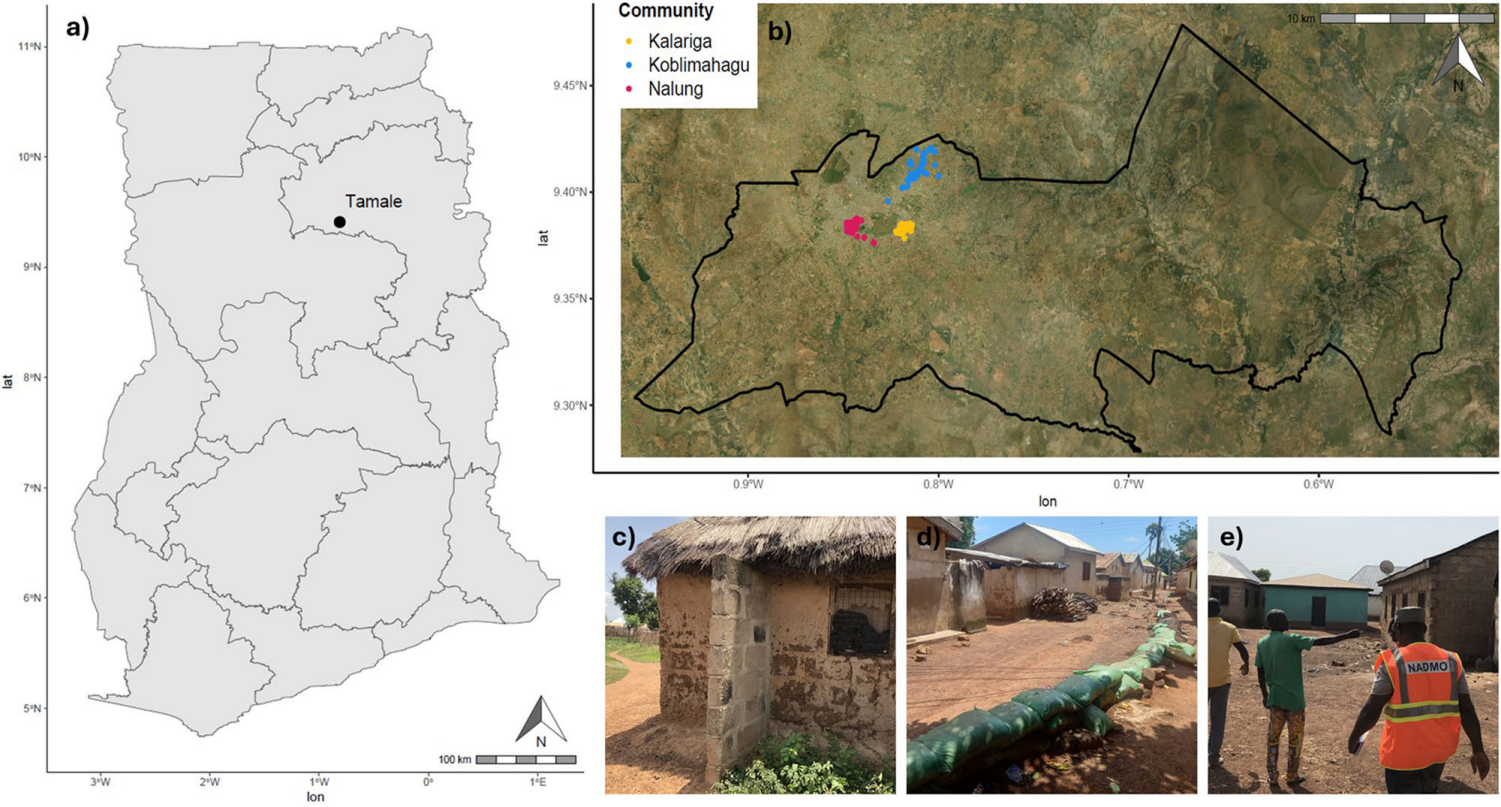
Details of the study location and methodology. **a** The location of Tamale within Ghana, showing regional boundaries. **b** The locations of the household surveys in three communities (Kalariga, Koblimahagu, and Nalung) within the Tamale Metropolitan Area, delineated by the black line. **c** Structural supports added to a mud brick house in Koblimahagu. **d** A sandbag embankment in Nalung. **e** Community members planning evacuation routes in Kalariga.

This study goes beyond previous work by testing the interactions between household characteristics and barriers to adaptation. Results highlight how different households have different barriers, suggesting that different policies and actions may be required to empower different households to adapt (Fig. 5). Although resources and time were not major barriers overall, they were important constraints for poor households with fewer members; critically, these households generally also had the lowest levels of adaptation (Fig. 2). This suggests that addressing resource constraints is essential for realising equitable citizen-led adaptation and reducing adaptation gaps, especially by targeting investment to the poorest households. Financing citizen-led adaptation could realise quick wins in risk reduction for the most vulnerable people; however, evidence suggests that this alone is likely limited in the magnitude of risk reduction it can achieve overall^32^. Our results (Figs. 2, 3) highlight the importance of community-led engagement with adaptation, including households with fewer members, as well as resource constraints. Understanding these limits (e.g., the threshold at which resources are no longer limiting) is important to effectively target barriers^39^. Beyond these limits, financing must be coupled with upskilling activities which equip individuals with the skills, knowledge, and networks necessary to enable citizen-led adaptation, as evidenced by the observed importance of flood education and social networks (Figs. 2, 4d, e).

In line with other studies^9,25,30,40^, we found a preference towards structural interventions as compared to behavioural (Fig. 1). This may be explained by differences in the effects of these interventions, whereby structural are more likely to reduce exposure (e.g., by blocking out flood water or elevating the house above it) whilst behavioural are more likely to reduce vulnerability^41^. Structural interventions may be perceived to reduce flood risk to near zero, which households value considerably, whilst behavioural may only limit the impacts and are therefore potentially a less attractive proposition^25,41^. Whilst the preferences of individuals are paramount to the long-term performance of adaptation interventions, practicing a combination of interventions is critical^13,41^. For example, structural interventions, especially on the household scale, may provide little protection against increasingly common large flood events^2,3,11^. During these events, behavioural adaptation, such as early warning and evacuation, will be essential to save lives^42^. We show that adaptive capacity is especially important in enabling behavioural interventions, particularly awareness and education components (Fig. 4). Therefore, increasing adaptive capacity is critical to supporting a more diverse and resilient suite of citizen-led adaptation strategies that can reduce risk during a large range of flood scenarios.

Although addressing barriers is important, doing so does not necessarily lead to increased practice of adaptation. Firstly, a hierarchy of barriers is likely where, if the primary barrier is addressed, another, or potentially several other, barrier(s) may still hinder the practicing of an intervention^43^. Our results hint at this phenomenon, as those for which access or expense did not present a barrier cited multiple other barriers instead. For example, people who had lived in the community for over 30 years cited access as a barrier half as much as the others but cited multiple reasons more than twice as much (Fig. 3d). Similar trends were also observed for those who had large families (Fig. 3a) or attended flood education previously (Fig. 3b). Secondly, value-action gaps are commonly observed, whereby the willingness and ability to act differs to actual behaviour^44^. For example, having access to flood education had considerable positive influence on practicing adaptation interventions (Fig. 2) and it was not considered unimportant (Fig. 1), but addressing the barriers to flood education (i.e., by improving skills, knowledge, and networks) might not result in increased participation. This research provides a useful starting point by identifying barriers that can be targeted in efforts to support citizen-led adaptation; however, it addresses an admittedly small component of the complex dynamics that determine behaviours and actions^38,45^.

In this study, the number of flood-risk adaptation interventions that households in Tamale practiced was placed into context with demographic indicators. In general, the willingness to practice further adaptation interventions is not diminished by having already practiced other interventions, suggesting that characterising the number of practiced interventions provides a suitable estimate of household level adaptation^46^. However, the risk reductions that adaptation provided, or indeed overall household risk (e.g., determined by hazard, exposure, and vulnerability), were not quantified; hence, the study does not account for the performance of interventions or the extent to which interventions were practiced (e.g., a house could be elevated by 0.2 m or 2 m and survey results would not capture the difference). Results can therefore be used to identify and address the barriers to citizen-led interventions, but they must be used in conjunction with other knowledge (e.g., effects of specific interventions) when applied in adaptation planning.

A recurring challenge in climate change adaptation research and practice is producing insights that are scalable and transferable across settings^6,47,48^. Citizen-led adaptation is determined by the socioenvironmental settings that it is developed within and applied to, as well as the experiences, values, and belief systems of the people who drive it^5^. Notwithstanding these important context dependencies, consistent patterns are beginning to emerge:

1. Citizen-led adaptation is occurring across the world, likely to a larger extent than previously assumed.
2. Without facilitation, citizen-led adaptation could potentially increase adaptation gaps, thereby exacerbating already profound inequalities in climate risk.
3. The primary barriers to citizen-led adaptation are access, knowledge, skills, and networks, but substantial heterogeneity exists between people and places.

Confidence in these key insights is invaluable for informing strategies aiming to support citizen-led adaptation, such as those that might be supported by the Fund for responding to Loss and Damage^49^. However, further work is needed to quantify the effects of citizen-led adaptation on risk, and in particular to identify its limits^32^. Improved understanding of the distribution and dependencies of existing adaptation interventions, including citizen-led adaptation, is essential for enabling integration into medium- and long-term adaptation strategies (e.g., national adaptation plans) and avoiding maladaptation^16,50,51^. Achieving equitable adaptation at the rate necessary to limit the worst impacts of climate change will require capitalising on every available opportunity, and citizen-led adaptation is one of the most promising^21,46^.

## Methods

Tamale, Ghana was chosen for the study because it is representative of many secondary cities in low- and middle-income countries^52^. It is one of the fastest growing cities in West Africa, with the population of the Tamale Metropolitan Area and its bordering municipalities having tripled in the past 25 years, putting strain on land availability and local services, such as city planning and water and sanitation services^23^. Both state and traditional (i.e., chiefs) authorities are responsible for the provision of public services in Ghana in a complex hybrid governance system that is common in Sub-Saharan Africa^53^. In this context, city-level climate change adaptation in Tamale is constrained by limited finance and complex governance systems, despite admirable aspirations^22,54^.

Whereas previously (before circa 1980) flooding was experienced infrequently if at all, many parts of Tamale are now subject to floods several times a year which is attributed to rapid urbanization and climate change^24,26^. Additionally, reports are common of inequitable storm drains that divert flood waters away from areas of high resource, status, or economic activity towards poor communities, overall representing maladaptation and increasing city-level flood risk^54,55^. Many of the city’s residents are highly vulnerable to flood impacts, with 21% living in multidimensional poverty and many living in mud houses (termed ‘water sugar’ houses) which are vulnerable to collapse during floods^56^. The combination of increasing frequency and magnitude of flooding, increasing populations in highly exposed areas, and the high vulnerability of many city residents to flood impacts is increasing the overall flood risk in Tamale considerably^54^.

This study is part of a larger programme of research and action in Tamale which adopts a co-production approach involving multiple stakeholders and bringing communities to the centre of the research process^57^. Workshops (*n* = 1), focus groups (*n* = 6), and interviews (*n* = 15) were conducted from November 2022 to May 2023 to inform the focus and design of the research, in which participants were identified using existing local networks and snowball sampling. Flooding was identified by participants as the primary climate risk that crosscuts all components of society and is therefore the focus of this research. Information from these discussions also determined the selection of adaptation interventions and barriers included in this current study. The co-production activities, along with existing literature (e.g.^6,58^,), informed the development of questionnaires which were piloted (*n* = 6) in September 2023 to ensure the salience and clarity of questions for participants.

301 questionnaires were conducted in person in October 2023 in three communities (Kalariga, Nalung, and Koblimahagu) in Tamale (Fig. 5). These communities experience annual flooding, at least in some areas, and represent a diversity of socioeconomic contexts. Community boundaries were delineated based on maps from the Tamale Metropolitan Assembly Spatial Planning Department. Within communities, surveyors aimed to recruit one participant (typically the household head) from every tenth house, representing between 5% and 10% of the total households in each community. If a household could not be recruited, surveyors moved onto the next household. Surveys were conducted by officers of the National Disaster Management Organisation (NADMO) and students of the University for Development Studies (Tamale campus), supervised by researchers. Data was recorded in English using Kobo toolbox^59^; additionally all surveyors were fluent speakers in Dagbani (the local language) and had attended a one-day training session, covering topics including proper translation of technical terms. Following quality control, 15 questionnaires were removed because several questions had been omitted or the location of the household had not been recorded, leaving a subset of relevant question responses from 286 questionnaires withheld for analysis.

The aim of the questionnaires was to determine the levels of practice of and barriers to household level adaptation to flooding in Tamale. Adaptation interventions were identified in collaboration with communities during the co-production process. We included interventions that participants knew were currently being practiced in their communities, and those that are driven by community members (i.e., citizen-led) and primarily occur on the household level (Table 1, Fig. 5). Interventions were characterized into structural (i.e., material changes to the environment, such as building houses on platforms) and behavioural (also known as non-structural; i.e., changes in the ways people interact with their environment, such as using early warning systems to make decisions). We used the number of interventions that are practiced as a measure of the level of adaptation of the household. Whilst this is considered generally indicative of access to adaptation, it is not necessarily related to risk reduction as it does not account for the performance of adaptation interventions.

**Table 1:** Common citizen-led adaptation interventions to flooding in Tamale, Ghana, as identified by communities, categorised, and described

Label	Category	Description
Community planning	Behavioural	Coming together to organise collective action, e.g., lobbying local governments (Fig. 5e).
Community practices	Behavioural	Coming together to improve shared spaces, e.g., drain clearing.
Drainage	Structural	Household level conduits that move water out of or away from the house.
Early warning systems (EWS)	Behavioural	Using early warning systems to inform decisions, e.g., to evacuate.
Emergency provisions	Behavioural	Storing essential items like food, water or money to help survive and/or recover from flooding.
Flood education	Behavioural	Engaging with awareness and education activities about flood risk and adaptation, e.g., in organised workshops or online.
Planting vegetation	Structural	Planting and/or managing plants (e.g., trees or grasses) to reduce flood generation.
Protect valuables	Behavioural	Storing valuables (e.g., money, documents, electronics) in flood-proof locations, e.g., elevated or watertight containers.
Raised elevation	Structural	Building the house on a platform to raise it above the typical flood water level.
Structural supports	Structural	Housing modifications to improve strength of the walls or roof of the house, e.g., block pillars or piers (Fig. 5c).
Walls and embankments	Structural	Erecting barriers between the house and the direction of flood water, e.g., sandbags or compound wall (Fig. 5d).

To explore the barriers to adaptation, we adopted the framework proposed by Oliver et al. (2023) which is based on a self-reporting of barriers, as described in Table 2^6^. Barriers from Oliver et al. (2023) were modified based on Tamale community priorities that were revealed during the co-production activities. For example, *‘I don’t have ownership/rights to do this’* was changed to *‘I don’t have access’* because whilst ownership or rights were not a concern in Tamale, knowing where or how to access or arrange adaptation interventions was a frequently cited issue. Additionally, *‘I don’t consider it to be my responsibility’* was included because this is a commonly cited barrier in top-down and community-based approaches which was also perceived in Tamale^5,34^. To complement this self-reporting of barriers approach, we also investigated the relations between household characteristics and levels of both practice of and barriers to adaptation. Household characteristics (Table 3) relate directly to survey responses and were selected based on similar examples in the literature and sense-checked in the co-production activities^47^. Together, the self-reported barriers and household characteristics allowed us to investigate if and how the barriers to adaptation vary for different groups of people.

**Table 2:** Self-reported barriers to adaptation after Oliver et al.^6^ and modified based on Tamale community input

Label	Description
No: It is too expensive	Does not practice intervention because prohibited by cost.
No: I don’t have time	Does not practice intervention because prohibited by time.
No: I don’t consider it to be my responsibility	Does not practice intervention because believes someone else is responsible.
No: I don’t consider it to be useful or important	Does not practice intervention because does not believe in its efficacy.
No: I don’t have access	Does not practice intervention because doesn’t know where or how to access or arrange it.
No: Multiple reasons	Does not practice intervention for multiple reasons (as selected or when >1 barrier).
No: Another reason	Does not practice intervention for a reason that is not a specified option.

**Table 3:** Household characteristics used as indicators of adaptive capacity, after Siders et al.^47^

Label	Description	Classes
Income	Household income per month in GH₵. Classes predefined.	<₵1000; ₵1000–₵2000; ₵2000–₵3000; ₵3000–₵4000; >₵4000
Income/person	Income per month (GH₵) divided by household size. Classes post-defined.	<₵1000; ₵1000–₵2000; ₵2000–₵3000; ₵3000–₵4000; >₵4000
Expenditure	Combined household expenditure per month (GH₵). Classes post-defined.	<₵1000; ₵1000–₵2000; ₵2000–₵3000; ₵3000–₵4000; >₵4000
Household size	Total number of people living in the household. Classes post-defined.	<3; 3–5; 6–10; 11–15; >16
Responsibility	Responsibility of the household and/or community in flood protection. Classes predefined.	No; Yes
Flood frequency	Frequency of flood occurrence directly around the household. Classes predefined.	>every 10 years; Approx. every 10 years; Approx. every 5 years; Most years; Not sure
Financial Setbacks	Experience of financial setbacks due to flooding. Classes predefined.	No; Yes – minor; Yes – major
Importance of resilience	Importance of building resilience to effectively cope with flood events in Tamale. Classes predefined.	Not important; Somewhat Important; Important; Very important
Flood education	Attended or accessed flood education and awareness activities or information. Classes predefined.	No; Yes
Early warning systems (EWS)	Uses early-warning systems to inform decisions. Classes predefined.	No; Yes
Access support	Access social services and/or support programs that can assist in preparing for or recovering from flood-related challenges. Classes predefined.	No; Yes
Education	Highest level of education of the household head. Classes predefined but amalgamated after.	No formal; Basic (inc. Islamic, primary, middle school, or technical); Secondary (inc. secondary or junior high school); Tertiary
External contributions	Receive external contributions (e.g., financial) from friends or relatives. Classes predefined.	No; Yes
Community cohesion	Observed changes to community relationships or cohesion during and after flood events. Classes predefined.	Yes, there was decreased community support and solidarity; No, there was no noticeable change; Yes, there was increased community support and solidarity
Help from friends	Seeking help from friends and/or relatives during or after flood events. Classes predefined.	No; Yes
Time in community	Number of years the household head has lived in this community. Classes post-defined.	<5; 5–10; 11–20; 21–30; >30 (years)

**Fig. 6:**
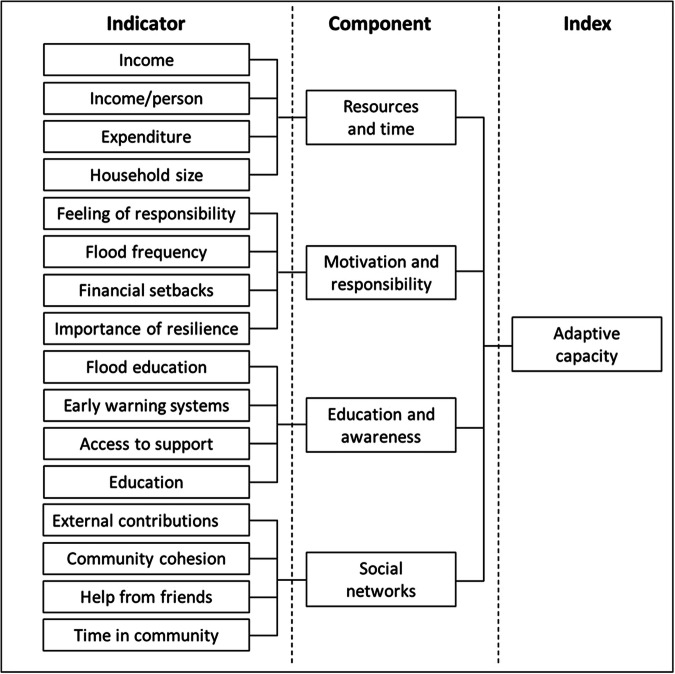
Schematic overview of the model of adaptive capacity. Four components contribute to the index of adaptive capacity: resources and time, motivation and responsibility, education and awareness, and social networks. Each component is defined by four indicators, as determined by responses to the household survey. The indicator-component-index model is commonly used in adaptive capacity research^60^.

To explore further the relations between household characteristics and level of adaptation, we calculated an index of adaptive capacity following the hierarchical index-component-indicator model used elsewhere (Fig. 6)^60^. The indicators were the household characteristics described in Table 3, which contain varying classes determined by either the possible questionnaire responses for categorical variables (e.g., Income: <₵1000, ₵1000–₵2000, ₵2000–₵3000, ₵3000–₵4000, >₵4000) or by expert judgement for continuous variables (e.g., Household size: <3, 3–5, 6–10, 11-, >16 people). The average percentage practice of all adaptation interventions by each class was calculated for every indicator. Where an indicator was also an adaptation intervention (i.e., flood education and early warning systems), these were removed to calculate the average uptake percentage of all adaptation interventions. This value was divided by the sum of all the classes in the indicator (i.e., classes in each indicator sum to 100%) and adjusted for the number of classes to normalize the indicators relative to one another. This provided a relative weighting for each class based on the observed relation between the participants in that class and their level of adaptation, whilst also ensuring that each indicator had the same potential influence on the component. Each participant was assigned a weighted value for each indicator based on their class in that indicator.

Indicators were categorized into four components of adaptive capacity based on the expert judgment of the co-production team, as opposed to using statistical methods, to ensure salience within a Tamale context^47^. For example, unlike many models for adaptive capacity, we included elements that pertain to personal attitudes and experience of risk (e.g., importance of resilience and flood frequency) and expectations about responsibility (e.g., feelings of personal responsibility) because such sentiment was evident in Tamale; these have been shown to be important in household level access to adaptation^35^. The four components were: (i) resources and time, (ii) motivation and responsibility, (iii) education and awareness and (iv) social networks (Fig. 6). The indicators in each component were summed to calculate a component value for each participant. The component values were summed to calculate a value of adaptive capacity for each participant. The scores for adaptive capacity overall and for each component separately were equally distributed into three classes (low, medium, and high) for analysis and visualization, i.e., Fig. 4. The developed index is valuable for shedding light on the relative influence of each component on both the levels of access and the barriers to interventions. It should be noted, however, that comparison with other indices is limited by context dependencies and a lack of consensus in the adaptive capacity field^47^.

### Ethics declaration

This study has been approved by the Department of Civil and Environmental Engineering at Imperial College London, UK (ID: 6571409), and by the Department of Geography and Resource Development, University of Ghana, Ghana (ID: 194/ 22–23). All participants provided informed consent. Every effort was made to ensure that activities were salient and as efficient as possible, e.g., activities were performed within Tamale communities. Activities were performed in accordance with the relevant guidelines.

## Supplementary information

Supplementary information

## Data Availability

The datasets generated and/or analysed during the current study are not publicly available due to their continued use in as-of-yet unsubmitted PhD theses but are available from the corresponding author on reasonable request.

## Code availability

The underlying code for this study (i.e., for production of the adaptive capacity index and visualizations) is not publicly available but may be made available to qualified researchers on reasonable request from the corresponding author.
