# Supplementary material for: Household-specific barriers to citizen-led flood risk adaptation
Source: NPJ Clim Action. Author manuscript; Available in PMC 2024 Dec 2. (PMC11588645; doi:10.1038/s44168-024-00198-y)
Supplement: Supplementary information [file EMS200898-supplement-Supplementary_information.docx]

**Supplementary Information**

**Household specific barriers to citizen-led flood risk adaptation**

Ben C. Howard*^1^, Cynthia A. Awuni^2,3^, Samuel Agyei-Mensah^1,2^, Lee D. Bryant^4^, Alexandra Collins^5^, Sandow Mark Yidana^6^, Gerald A.B. Yiran^2^, Wouter Buytaert^1^

1. Dept. of Civil and Environmental Engineering, Imperial College London, UK
2. Dept. of Geography and Resource Development, University of Ghana, Ghana
3. Tamale Technical University, Department of Hospitality and Tourism Management, Tamale, Ghana
4. Dept. of Architecture and Civil Engineering, Centre for Climate Adaptation and Environment Research (CAER), University of Bath, UK
5. Centre for Environmental Policy, Imperial College London, UK
6. Dept. of Earth Science, University of Ghana, Accra, Ghana

* Corresponding author.

Corresponding author email address: ben.howard@imperial.ac.uk

Presented below is the template of the household level questionnaire deployed in Tamale, Ghana, in September 2023. Responses to some questions were used in this study.

**Socio-Demographic**

1. Sex of respondent
   1. Male
   2. Female
   3. Non-binary/Other (Please specify: __________)
2. Age of respondent...............................................................
3. Marital status
   1. Single
   2. Married
   3. Divorce
   4. Living together
   5. Widowed
   6. Other, please specify……………………………
4. Highest level of education of the respondent
   1. No formal education
   2. Primary education
   3. Middle school
   4. Junior High School
   5. Secondary education
   6. Tertiary education
   7. Technical/Vocational education
   8. Islamic education
   9. Other, please specify………………………….
5. What is the average monthly income of your household (all income combined)?

*This*

- 1. Less than ₵1,000
  2. ₵1,000 - ₵2,000
  3. ₵2,001 - ₵3,000
  4. ₵3,001 - ₵4,000
  5. More than ₵4,000

1. Do you have savings?
   1. Yes
   2. No
   3. Not sure
2. How many people in your household contribute economically (e.g. are employed)?

……………………………………….

1. Which of the following economic activities do you engage in?

01. Agriculture (inc. fishing)

02. Government work

03. business

04. Artisanal, manufacture, and construction (masonry, carpentry, tailoring, hairdressing, mechanics etc.)

05. Informal labour or petty trading

06. Other ………………

1. On a scale of 1 to 5, how are these activities (i.e. from question 8) affected by flooding (where 1 is not impacted at all and 5 is severely impacted)?

……………………………… ……………………….

……………………………… ……………………….

……………………………… ……………………….

……………………………… ……………………….

……………………………… ……………………….

- I don’t know.

1. What are the primary sources of income for your household? (Select all that apply)
   1. Salary or wages
   2. Investments and savings
   3. Rental income or business
   4. Social assistance, government benefits, or pension
   5. Informal work or petty trading
   6. Donations
   7. Other (please specify)
2. Five years ago, did you have the same sources of income?
   1. Yes
   2. No
3. How long have you lived in this community?...........................................................

**Housing**

1. How long has your household been residing in the current location?

*i.e. how long have they lived in this exact spot. If they have moved house but stayed in the same community, then record the date they moved.*

- 1. Less than 1 year
  2. 1-5 years
  3. 6-10 years
  4. More than 10 years

1. Residential status
   1. Own House
   2. Rent-paid.
   3. Family House
   4. Perching (paying rent)
   5. Squatting
   6. Rent-free
   7. Other, please specify……………………………………………………
2. Type of dwelling unit occupied by the household.
3. Flat/Apartment
4. Detached house
5. Semi-detached
6. Compound House
7. Single Room
8. Uncompleted building
9. Kiosk/Container (improvised home)
10. Other, please specify…………………………………
11. Approximately when was this house constructed?
    1. After 2020
    2. 2010 - 2020
    3. 2000 – 2010
    4. Before 2000
12. Type of Family
13. Nuclear (monogynous)
14. Nuclear (polygynous)
15. Joint/Extended
16. What is/are the age bracket of people in the household?
17. below 18 years: ……………….
18. 18 – 35 years: ……………….
19. 36 -59 years: ……………….
20. 60 years and above: ……………….
21. Total dependents: ……………….
22. Number of dependents in education (inc. university) ………………….
23. Do any persons with disabilities live in your household?
    1. Yes
    2. No
24. Does anyone in your household have a chronic health condition that requires regular medication or medical attention?
    1. Yes, one or more household members
    2. No, none
25. Do you receive external contributions to the household, for example from friends or relatives?
    1. Yes
    2. No
26. What kind of contribution do they give you? (*tick all that apply*)
    1. Food and water
    2. Money
    3. Clothes
    4. Building materials
    5. Agricultural input
    6. Other ……………………………………..
27. Does it increase when there is a flood?
    1. Yes
    2. No

**Flood Hazard**

1. Since you have lived in this community, what changes in temperature have you observed? *Note that this refers to long term changes, i.e. >10 years.*
   1. Increases in temperature
   2. Decreases in temperature
   3. No change in temperature
   4. Not sure
   5. Other ……
2. Since you have lived in this community, what changes to the length of the rainy season have you observed? *Many respondents will be not sure, and that is a valid answer.*
3. Shorter length of rainy season
4. Longer length of rainy season
5. No change
6. Not sure
7. Other……………………
8. Since you have lived in this community, what changes to the frequency of rainfall have you observed? *Many respondents will be not sure, and that is a valid answer.*
9. Reduced frequency of rainfall
10. Increased frequency of rainfall
11. No change
12. Not sure
13. Other……………………
14. Since you have lived in this community, what changes to the intensity (heavy or light) of rainfall have you observed? *Many respondents will be not sure, and that is a valid answer.*
15. Reduced intensity of rainfall
16. Increased intensity of rainfall
17. No change
18. Not sure
19. Other……………………
20. What will you attribute the changes to the rainfall? (*tick all that apply*)
21. Cutting down of trees
22. Bush burning
23. Climate change
24. Gods will
25. Natural variability
26. Other……………………….
27. On average, how often do floods occur directly around your household? *i.e. in or surrounding the household or property.*
28. Most years
29. At least every 5 years
30. At least every 10 years
31. Les
32. Never
33. Not sure
34. Other (specify) ………….
35. How has flooding changed directly around your household since you moved to this house? (*tick all that apply*) *This refers to flood water in or directly around the household.*
    1. Not at all.
    2. Increase in frequency.
    3. Decrease in frequency.
    4. Increase in flood water depth.
    5. Decrease in flood water depth.
    6. Other…………………………..
36. How often does the flood water enter your household? *This means that the flood water is inside the house, i.e. the areas where people live (sleep, cook, eat).*
37. Most years
38. At least every 5 years
39. At least every 10 years
40. Less than every 10 years
41. Never
42. Not sure
43. Other (specify) ………….
44. How long does the flood usually last?
45. 0 to 1 day
46. 2 to 3 days
47. 4 to 5 days
48. 1 week or more
49. In the last flood that occurred, what possessions and property were damaged in your household? (*tick all that apply*)?
50. Building.
51. Access road.
52. Services (water, sanitation, electricity).
53. Provisions (food, medicines).
54. Valuables (electronics, jewellery, furniture).
55. Other ………………………
56. What are your greatest fears about flooding? (*Tick all that apply*)
57. Personal safety
58. Safety of relatives
59. Household’s goods and possessions
60. Building collapse
61. Uncertainty of flood occurrence
62. Others (Specify)…………………………………………………………………

**Vulnerability Assessment**

1. What kind of vehicle do you own?
   1. Car
   2. Motorbike
   3. Other…………………..
   4. None
2. If so, is it insured against flood damage?
3. Yes
4. No
5. Have you experienced minor, major or none at all financial setbacks due to previous flood events?
6. Yes, major financial setbacks
7. Yes, minor financial setbacks
8. No, no financial setbacks
9. Do you have an emergency savings fund that can cover immediate expenses in case of a flood?
10. Yes, we have an emergency fund
11. No, we do not have an emergency fund
12. Not sure
13. How satisfied are you with the response of first responders (e.g., NADMO, police, fire, rescue teams) during past flood events?
14. Very satisfied
15. Somewhat satisfied
16. Not satisfied
17. No experience with first responders
18. Do you have insurance coverage (e.g., flood insurance, homeowners/renters insurance) that could help with flood-related losses?
19. Yes, for both flood insurance and homeowners/renters insurance
20. Yes, for flood insurance only
21. Yes, for homeowners/renters insurance only
22. No insurance coverage
23. Not sure
24. Does your household have access to healthcare services, including medical insurance?
25. Yes, we have access to healthcare services and insurance
26. No, limited access to healthcare services
27. Do you have access to government social services and/or NGO support programs in that can assist in preparing for or recovering from flood-related challenges?
28. Yes, we are aware and have access
29. No, not aware or limited access
30. Have you or anyone in your household considered or experienced temporary or permanent relocation due to flood-related concerns?
31. Yes, we have considered or experienced relocation
32. No, relocation has not been considered

**Flood Perception and Adaptation**

1. During the flood event, what actions do you take to manage the immediate impact of the flood? (*Tick all that apply*)
2. Evacuated to a safer location
3. Sought help from neighbours or community members
4. Sought help from friends and relatives
5. Erect temporary barriers or push back against water
6. Stayed in my home and waited for the flood to recede
7. Other (Please specify)………………………………………
8. Who is responsible for Flood protection? (*Tick all that apply*)
9. Individual households
10. Government
11. Flood management experts
12. Company
13. Community
14. Public (outside of community)
15. NGO
16. Did the flood lead to any changes in community relationships or cohesion?
17. Yes, there was increased community support and solidarity
18. Yes, there was decreased community support and solidarity
19. No, there were no noticeable changes
20. How resilient do you think your community is to future flood events? *Resilience means ability to cope with, stand up to, or recover from the flooding.*
21. Very resilient
22. Somewhat resilient
23. Not resilient at all
24. On a scale of 1-5 (1 not at all, 5 very), how important is it to build resilience in Tamale to effectively cope with flood events?

………………………………………………………..

1. Do you presently use early warning systems or weather information to inform your actions?

Yes.

*If yes, where did you access this information?*

- TV or radio.
- Online.
- NADMO
- Other (please specify)……………….

**No.***If no, why do you not to do this? (tick all that apply)*

- I don’t have access.
- It is too expensive.
- I don’t consider it to be useful or important.
- I don’t have time to do this.
- Multiple reasons.
- Another reason.
  ______________________

1. Have you attended or accessed flooding educational and awareness activities or information?

Yes.

*If yes, where did you access this information?*

- NGO community activity.
- Government community activity.
- Self-driven (e.g. online or books).
- Other (please specify)……………….

No.

*If no, why do you not to do this? (tick all that apply)*

- I don’t have access.
- It is too expensive.
- I don’t consider it to be useful or important.
- I don’t have time to do this.
- I don’t consider it to be my responsibility.
- Multiple reasons.
- Another reason.
  ______________________

1. Do you receive relief items (emergency provisions) following a flood?

Yes

*Who are they supplied by?*

- Government (including NADMO)
- NGOs
- Relatives or friends
- Community members
- Other (please specify)……………………..

*Are they always sufficient for your needs?*

- Yes
- No

*How long do they take to be delivered?*

- <1 day
- 1-3 days
- 3-7 days
- >7 days

No
*Do you have access to them?*

- Yes
- No

*If yes, why do you not use them?*

__________________________

Unsure

1. How do you think the storm drain network affects flooding for your household?

- They improve the flood situation.
- The make the flooding worse.
- They have no effect on the flooding.
- Unsure.

1. Estimate in meters how close the nearest storm drain (i.e. gutter) is to your house?

………………………………………………..

*Researcher use only: this section refers to bottom-up adaptation approaches in the community. All questions should follow the responses below.*

The following questions are about your household’s access to services and interventions in the community.

1. Do you engage in community practices such as drain clearing or solid waste management to reduce flooding?

Yes.

No.

*If no, why do you not to do this? (tick all that apply)*

- I don’t have access.
- It is too expensive.
- I don’t consider it to be useful or important.
- I don’t have time to do this.
- I don’t consider it to be my responsibility.
- Multiple reasons.
- Another reason.
  ______________________

1. Do you engage in community planning and initiatives to address flooding, e.g. emergency response planning or lobbying local government?

Yes.
No.

*If no, why do you not to do this? (tick all that apply)*

- I don’t have access.
- It is too expensive.
- I don’t consider it to be useful or important.
- I don’t have time to do this.
- I don’t consider it to be my responsibility.
- Multiple reasons.
- Another reason.
  ______________________

*Researcher use only: this section refers to bottom-up adaptation approaches in the household. All questions should follow the responses below.*

The following questions are about the ways you cope with flooding within your household.

1. Have you added supports (e.g. piers, pillars, foundation walls) to increase the resilience of your house to flooding?

Yes.
No.

*If no, why do you not to do this? (tick all that apply)*

- I don’t have access.
- It is too expensive.
- I don’t consider it to be useful or important.
- I don’t have time to do this.
- I don’t consider it to be my responsibility.
- Multiple reasons.
- Another reason.
  ______________________

1. Have you raised the elevation of your house or property (e.g. platform or gravel)?

Yes.
No.

*If no, why do you not to do this? (tick all that apply)*

- I don’t have access.
- It is too expensive.
- I don’t consider it to be useful or important.
- I don’t have time to do this.
- I don’t consider it to be my responsibility.
- Multiple reasons.
- Another reason.
  ______________________

1. Have you erected barriers (e.g. wall, compound, embankment) around some or all of your household?

Yes.
No.

*If no, why do you not to do this? (tick all that apply)*

- I don’t have access.
- It is too expensive.
- I don’t consider it to be useful or important.
- I don’t have time to do this.
- I don’t consider it to be my responsibility.
- Multiple reasons.
- Another reason.
  ______________________

1. Have you constructed drainage (e.g. sandbag channels) in and/or around your household?

Yes.
No.

*If no, why do you not to do this? (tick all that apply)*

- I don’t have access.
- It is too expensive.
- I don’t consider it to be useful or important.
- I don’t have time to do this.
- I don’t consider it to be my responsibility.
- Multiple reasons.
- Another reason.
  ______________________

1. Have you planted trees or vegetation to help with flooding?

Yes.
No.

*If no, why do you not to do this? (tick all that apply)*

- I don’t have access.
- It is too expensive.
- I don’t consider it to be useful or important.
- I don’t have time to do this.
- I don’t consider it to be my responsibility.
- Multiple reasons.
- Another reason.
  ______________________

1. Do you keep/preserve additional provisions (e.g. water, food) in case of a flood?

Yes.
No.

*If no, why do you not to do this? (tick all that apply)*

- I don’t have access.
- It is too expensive.
- I don’t consider it to be useful or important.
- I don’t have time to do this.
- I don’t consider it to be my responsibility.
- Multiple reasons.
- Another reason.
  ______________________

1. Do you keep/protect possession high up or in boxes in case of flood?

Yes.
No.

*If no, why do you not to do this? (tick all that apply)*

- I don’t have access.
- It is too expensive.
- I don’t consider it to be useful or important.
- I don’t have time to do this.
- I don’t consider it to be my responsibility.
- Multiple reasons.
- Another reason.
  ______________________

Researcher observations:

*What material are the walls of the house constructed from?*

- Concrete blocks
- Mud
- Mud blocks
- Metal
- Wood
- Multiple
- Unsure
- Other ……………………………

*What adaptation features can you see? (tick all that apply)*

- Platform
- Doors higher up
- Piers or pillars
- Sandbag bank
- Embankment
- Compound wall
- Door barriers
- Platform
- Elevation increase

Other(s):______
